# Molecular detection of hemoplasmas in domestic cats from different Brazilian regions

**DOI:** 10.1590/S1984-29612025035

**Published:** 2025-06-16

**Authors:** Clara Morato Dias, Kamila Pinheiro Francisco, Gabriela Veiga Oliveira, João Vitor dos Santos Alves da Silva, Eliz Oliveira Franco, Daniel Antônio Braga Lee, Dália Monique Machado Ribeiro, Maria Eduarda Chiaradia Furquim, Mayra Araguaia Pereira Figueiredo, Rosangela Zacarias Machado, Marcos Rogério André

**Affiliations:** 1 Laboratório de Bioagentes Transmitidos por Vetores, Departamento de Patologia, Reprodução e Saúde Única, Faculdade de Ciências Agrárias e Veterinárias – FCAV, Universidade Estadual “Júlio de Mesquita Filho” – UNESP, Jaboticabal, SP, Brasil; 2 Laboratório e Hemocentro Veterinário – HEMOLABVET, Ribeirão Preto, SP, Brasil; 3 Laboratório de Parasitologia, Entomologia e Biologia Molecular Aplicada à Saúde Única, Universidade Federal de Rondônia – UNIR, Rolim de Moura, RO, Brasil

**Keywords:** '*Candidatus* Mycoplasma haemominutum', *Mycoplasma* spp., qPCR, feline hemoplasmosis, '*Candidatus* Mycoplasma haemominutum', *Mycoplasma* spp., qPCR, hemoplasmose felina

## Abstract

Domestic cats can be parasitized by *Mycoplasma haemofelis*, ‘*Candidatus* Mycoplasma haemominutum’, and ‘*Candidatus* Mycoplasma turicensis’. Although the molecular occurrence of hemoplasmas in domestic cats has been investigated in the five geographical regions of Brazil, no studies have been conducted in the states of Rondônia and Minas Gerais to date. The present work aimed to investigate the molecular occurrence of hemoplasmas in cats from four Brazilian states in the Northern, Central-Western and Southeastern regions of the country. Among 486 blood samples – 80 from Rondônia (RO), 100 from Goiás (GO), 155 from Minas Gerais (MG), and 151 from São Paulo (SP) – submitted to the endogenous *gapdh* gene-based PCR, 94.44% (459/486) were positive, of which 1.96% (9/459; 1 from RO, 2 from GO, 5 from MG, and 1 from SP) were positive in the qPCR assay for hemoplasmas based on the 16S rRNA gene. In the phylogenetic analyses, the obtained 16S rRNA (two genotypes) and 23S rRNA (three genotypes) sequences were positioned together with ‘*Candidatus* Mycoplasma haemominutum’. The present study showed the first molecular evidence of infection by hemoplasmas in cats from MG and RO, contributing to a better understanding of the epidemiology of feline hemoplasmosis agents in Brazil.

## Resumo

Gatos domésticos podem ser parasitados por *Mycoplasma haemofelis, ‘Candidatus* Mycoplasma haemominutum’ e *‘Candidatus* Mycoplasma turicensis’. Embora a ocorrência molecular de hemoplasmas em gatos domésticos tenha sido investigada nas cinco regiões geográficas do Brasil, nenhum estudo foi conduzido nos estados de Rondônia e Minas Gerais até o momento. O presente trabalho teve como objetivo investigar a ocorrência molecular de hemoplasmas em gatos de quatro estados brasileiros nas regiões Norte, Centro-Oeste e Sudeste do país. Dentre 486 amostras de sangue – 80 de Rondônia (RO), 100 de Goiás (GO), 155 de Minas Gerais (MG) e 151 de São Paulo (SP) – submetidas à PCR baseada no gene endógeno *gapdh*, 94,44% (459/486) foram positivas, das quais 1,96% (9/459; 1 de RO, 2 de GO, 5 de MG e 1 de SP) foram positivas no ensaio de qPCR para hemoplasmas baseado no gene 16S rRNA. Nas análises filogenéticas, as sequências 16S rRNA (2 genótipos) e 23S rRNA (3 genótipos) obtidas foram posicionadas juntamente com *‘Candidatus* Mycoplasma haemominutum’. O presente estudo mostrou a primeira evidência molecular de infecção por hemoplasmas em gatos dos estados de Minas Gerais e Rondônia, contribuindo para um melhor entendimento da epidemiologia dos agentes da hemoplasmose felina no Brasil.

## Introduction

Hemoplasmas (Mollicutes: Mycoplasmataceae) are cell wall-lacking small epicellular bacteria that infect the surface of erythrocytes of vertebrate hosts (Razin et al., 1998). These bacteria have already been reported in different species of domestic and wild mammals, including pigs, cattle, sheep, bears, bats, rodents, dogs, and cats (Harasawa et al., 2015; Mesquita et al., 2021; Tasker, 2022). While the transmission of such agents via aggressive interactions and blood transfusion is well documented, vector transmission by fleas, ticks and mosquitoes is still poorly elucidated, being restricted to studies that detected DNA from hemoplasmas resulting from the blood meal of these ectoparasites (Tasker, 2022; Moore et al., 2024). Furthermore, hemoplasma infections are important causes of anemia in felines (Tasker, 2022; Moore et al., 2024).

Domestic cats can be parasitized by three hemoplasma species, namely *Mycoplasma haemofelis* (Mhf), ‘*Candidatus* Mycoplasma haemominutum’ (CMhm) and ‘*Candidatus* Mycoplasma turicensis’ (CMt), the former considered the most pathogenic species (Tasker, 2022; Moore et al., 2024). Mhf is responsible for causing severe extravascular and occasionally intravascular hemolytic anemia in immunocompetent cats without other comorbidities (Willi et al., 2005; Tasker, 2022; Hoseinpoor et al., 2024). CMhm, in its turn, causes clinical anemia in cats with neoplasms, immunosuppressed, or infected with the feline leukemia virus (FeLV) (Rosa Maciel et al., 2023). On the other hand, CMt infection can result in a small reduction in the number of erythrocytes experimentally, but rarely causes clinical anemia (Willi et al., 2005; Tasker, 2022).

Feline hemoplasmosis is often associated with old, male, mixed-breed cats with outdoor access (Kellner et al., 2018; Tasker, 2022). Díaz-Regañón et al. (2018) showed a significant relationship between some species of hemoplasma and retroviruses, especially the feline immunodeficiency virus (FIV) (Attipa et al., 2017; Rosa Maciel et al., 2023). Due to failures in the in vitro cultivation of hemotropic *Mycoplasma* spp., as well as the low sensitivity of cytological tests and blood smears for detecting these agents, molecular diagnosis by Polymerase Chain Reaction (PCR) is the most suitable technique for diagnosing hemoplasmosis (Tasker, 2022).

Different research groups have molecularly detected hemoplasmas in domestic cats in various countries, such as Iran (CMhm: 10.5%; Mhf: 2.2%; CMt: 0.6%) (Hoseinpoor et al., 2024), China (CMhm: 3.4%; Mhf: 0.9%; CMt: 1.2%) (Zhang et al., 2021), Germany (CMhm: 8.76%; Mhf: 0.41%) (Bergmann et al., 2016), Portugal (CMhm: 41.56%; Mhf: 12.81%; CMt: 1.25%) (Martínez-Díaz et al., 2013), Albania (CMhm: 21.9%; Mhf: 10.3%; CMt: 5.5%) (Silaghi et al., 2014), Spain (CMhm: 9.9%; Mhf: 3.7%; CMt: 0.5%) (Díaz-Regañón et al., 2018), Romania (CMhm: 72.7%; Mhf: 27.3%) (Imre et al., 2020), Italy (CMhm: 12.3%; Mhf: 9.4%; CMt: 4.8%) (Ravagnan et al., 2017), South Africa (CMhm: 38%; Mhf: 15%; CMt: 26%) (Willi et al., 2006), Angola (CMhm: 7.5%; Mhf: 1.5%) (Mesquita et al., 2021) and the United States (*Mycoplasma* spp.: 13.69%) (Manvell et al., 2021). In Brazil, the molecular occurrence of hemotropic *Mycoplasma* spp. in domestic cats has been investigated in the five geographical regions of the country (Bortoli et al., 2012; Braga et al., 2012; Miceli et al., 2013; André et al., 2014; Santis et al., 2014; Santos et al., 2009; Raimundo et al., 2016; Munhoz et al., 2018; Pedrassani et al., 2019; Santos et al., 2021; Carvalho et al., 2024). However, no studies have been conducted in the states of Rondônia and Minas Gerais to date, and few studies have been conducted in the states of São Paulo and Goiás.

Although traditionally recognized for their veterinary significance, the zoonotic potential of hemoplasmas species has gained increasing attention in recent years, particularly in immunocompromised humans (Sykes et al., 2010). *‘Candidatus* M. haematohominis’, *‘Candidatus* M. haematoparvum’, *M. haemofelis*, *Mycoplasma haemocanis*, *Mycoplasma suis,* and *Mycoplasma ovis* have been molecularly detected in humans (Yuan et al., 2009; Sykes et al., 2010; Steer et al., 2011; Maggi et al., 2013, Hattori et al., 2020; Kmetiuk et al., 2025). For instance, Mhf has already been reported in a patient coinfected with HIV virus and *Bartonella henselae* in Brazil (Santos et al., 2008). CMt has been described in a patient with hemolytic anemia and pyrexia in the United States (Steer et al., 2011). Such reports imply constant epidemiological surveillance of feline hemoplasmosis in domestic cats and a One Health approach to the zoonotic risks of these agents. The present work aimed to investigate the molecular occurrence and genetic diversity of hemoplasmas in blood samples from cats from four Brazilian states in the Northern, Central-Western and Southeastern regions of the country.

## Material and Methods

### Study area and sampling

**Figure 1:**
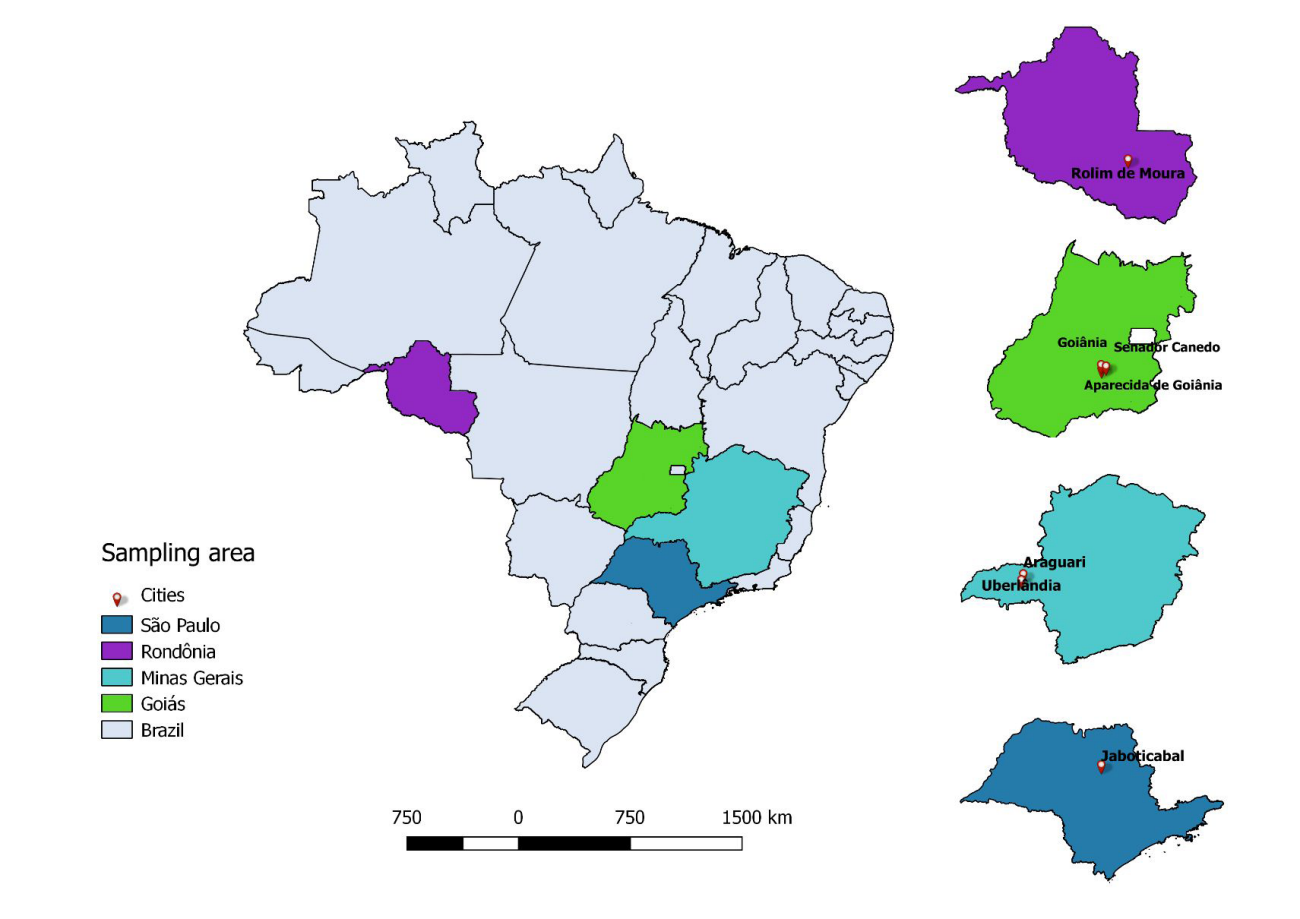
Map generated by QGIS 2.18.2.8, showing the states of Brazil (in different colors) and the location of cities by state where cats were sampled in the present study.

A total of 486 cat blood samples were collected in the states of Goiás (n=100), Minas Gerais (n=155), Rondônia (n=80) and São Paulo (n=151) (Figure 1) through partnerships with veterinary clinics and autonomous animal protection shelters, and during castration campaigns promoted by the Centro de Castração da Associação Protetora dos Animais, located in the Faculdade de Ciências Agrárias e Veterinárias (FCAV/UNESP, Jaboticabal, São Paulo), as well as the Projeto de Controle Populacional Animal developed by the Universidade Federal de Uberlândia (UFU, Uberlândia, Minas Gerais) and the Centro de Zoonoses in the same city (Furquim et al., 2021; André et al., 2022). The procedures and management involving domestic cats were approved by the “Comissão de Ética no Uso de Animais” (CEUA) from FCAV/UNESP, under protocol numbers 012017/17 and 5439/20.

Approximately 500 μL of blood from each feline was collected in sterilized tubes with EDTA anticoagulant (ethylenediaminetetraacetic acid). Then, samples were stored in cryotubes, free of RNAse and DNAse, and transported in a liquid nitrogen cylinder to the Vector Borne-Bioagents Laboratory (VBBL) (UNESP/FCAV – Campus Jaboticabal, SP). The samples were kept at -70 °C until further processing.

### DNA extraction

DNA extraction from cat blood samples was performed using the Biopur Mini Spin Plus kit (Mobius Life Sciences, Pinhais, Paraná, Brazil), following the manufacturer's recommendations. The 260/280 and 260/230 ratios and DNA concentration were measured using a NanoDrop spectrophotometer device (Thermo Scientific, Waltham, MA, USA).

### Conventional PCR (PCR) for the endogenous mammalian glyceraldehyde-3-phosphate dehydrogenase (*gapdh*) gene

PCR assays were performed to verify the absence of PCR inhibitors in DNA samples extracted from feline blood through amplification of the endogenous mammalian *gapdh* gene (Birkenheuer et al., 2003). Samples negative in this protocol were not used in further molecular analyzes.

### Quantitative real-time PCR (qPCR) based on the 16S rRNA gene from hemoplasmas

DNA samples positive for the endogenous gene were subjected to a qPCR to detect and quantify the number of copies of a fragment of the 16S rRNA gene from hemoplasmas per microliter, as described by Willi et al. (2009) (Table 1). To construct the standard curve for each reaction, serial dilutions were performed at different concentrations (1.0 × 10^7^ to 1.0 × 10^0^ copies) of a GBLOCK that encodes a 259 bp fragment of the 16S rRNA gene from *Mycoplasma haemofelis* (pIDTSMART; Integrated DNA Technologies, Coralville, Iowa, USA). DNA samples were evaluated in duplicates and, when the differences in Cq values ​​were greater than 0.5, they were re-tested in triplicates. Ultrapure water free of DNAses and RNAses (Promega, Madison, Wisconsin, USA) was used as a negative control in all reactions. The qPCR assays were conducted in the C1000-CFX96 thermocycler (BioRad®, Hercules, California, USA). The amplification efficiency (E) was calculated according to the slope of the standard curve using the formula (E= 10^-1/slope^) (Bustin et al., 2009). The data generated were visualized and stored in the CFX manager program (BioRad®, Hercules, California, USA).

**Table 1:** Description of the PCR assays used in this study based on the mammalian *gapdh* gene, and 16S rRNA and 23S rRNA of hemoplasmas.

**Molecular Marker**	**Primers**	**Thermal conditions**	**Reagent concentrations**	**Amplicon size (bp)**	**Reference**
*gapdh*	GAPDH-F (5’- CCTTCATTGACCTCAACTACAT-3’)	95 °C for 5min; 35 cycles of 95 °C for 15s, 50 °C for 30s and 72 °C for 30s; 72 °C for 5min	5 μL of sample DNA	450	Birkenheuer et al. (2003)
	
	GAPDH-R (5’- CCAAAGTTGTCATGGATGACC -3’)		0.2 mM dNTPs		
	
			0.4 μM of each primer		
	
			3.0 mM MgCl_2_,		
	
			1.25 U Taq Platinum DNA Polimerase		
16S rRNA	SYBR_For (5'-AGCAATRCCATGTGAACGATGAA-3')	50 °C for 2min, 95 °C for 10min; 40 cycles of 95 °C for 15s, 60 °C for 1min	1 μL of sample DNA	259	Willi et al. (2009)
		
	SYBR_Rev2 (5'-GCTGGCACATAGTTAGCTGTCACT-3')	Melting curve: 95 °C for 15s, 60 °C for 20s, increase from 60 °C to 95 °C for 20min and 95 °C for 15s	0.3 μM of each primer		
		
			12.5 μL of 2 x SYBR green PCR master mix (Applied Biosystems)		
		
16S rRNA	First round:	95 °C for 4min; 40 cycles of 95 °C for 15s, 60 °C for 30s and 72 °C for 15s; 72 °C for 7min	4 mM MgCl_2_	1,107	Di Cataldo et al. (2020) and Millán et al. (2019)
	HemoF1 (5'-AGAGTTTGATCCTGGCTCAG-3')				
			1 µM of each primer		
	HemoR2 (5'-TACCTTGTTACGACTTAACT-3')				
			0.2 mM dNTPs		
	Second round:				
	HemoF1 (5'-AGAGTTTGATCCTGGCTCAG-3')		1X buffer solution		
	
	HemoR2 (5'-TACCTTGTTACGACTTAACT-3')		1.25 U of Taq GoTaq^®^ G2 Hot Start Taq Polymerase (Madison, Wisconsin, USA)		
	
			First reaction: 5 μL of sample DNA		
	
			Second reaction: 1 μL of amplicon		
23S rRNA	23S_HAEMO_F (5′-TGAGGGAAAGAGCCCAGAC-3′)	94 °C for 3min; 35 cycles of 94 °C for 30s, 54 °C for 30s and 72 °C for 60s; 72 °C for 10min	5 µL of sample DNA	800	Mongruel et al. (2020)
	
	23S_HAEMO_R (5′-GGACAGAATTTACCTGACAAGG-3′)		1X buffer solution		
	
			1.5 mM MgCl_2_		
	
			0.2 mM dNTP		
	
			0.4 mM of each primer		
	
			2.5 U of Taq Platinum DNA Polymerase (Invitrogen, Carlsbald, California, USA)		

### Molecular characterization of hemoplasmas based on the 16S rRNA gene

DNA samples positive in qPCR were subjected to a semi-nested PCR (snPCR) assay amplifying an 1,107 bp fragment of the 16S rRNA gene described by Di Cataldo et al. (2020) to additional molecular characterization (Table 1). In all reactions, *Mycoplasma ovis* DNA was used as a positive control (Mongruel et al., 2020), while ultra-pure water free of DNAses and RNAses (Promega, Madison, Wisconsin, USA) was used as a negative control.

### Molecular characterization of hemoplasmas based on the 23S rRNA gene

Samples positive in qPCR were subjected to a conventional PCR assay (PCR) described by Mongruel et al. (2020), amplifying an 800 bp fragment of the 23S rRNA gene (Table 1). In all reactions, a sample positive for *Mycoplasma ovis* (Mongruel et al., 2020) was used as a positive control, while ultra-pure water free of DNAses and RNAses (Promega, Madison, Wisconsin, USA) was used as a negative control.

### Agarose gel electrophoresis

The products amplified in the snPCR and PCR assays were subjected to horizontal electrophoresis in a 1.0% agarose gel stained with Ethidium Bromide (0.5 μL/mL) in TBE pH 8.0 running buffer (44.58 M Tris-base; 0.44 M boric acid; 12.9 mM EDTA), being subjected to 90 V/ 150 mA for 60 minutes. A 100 base pair molecular marker (Life Technologies - Invitrogen, Carlsbad, CA, USA) was used and the results were visualized and analyzed using an ultraviolet light transilluminator (ChemiDoc MP Imaging System, BioRad). The data generated was stored in the Image lab program, BioRad.

### Purification, sequencing and phylogenetic analysis

The amplicons obtained in the snPCR and PCR assays for the 16S rRNA and 23S rRNA genes from hemoplasmas were purified using the Wizard SV Gel and PCR Clean-Up System kit (Promega, Madison, Wisconsin, USA). The purified products were sequenced using an automated technique based on the Sanger method (Sanger et al., 1977). Sequencing was conducted by the Centro de Recursos Biológicos (CREBIO) at FCAV/UNESP.

The Phred-Phrap version 23 program (Ewing & Green, 1998; Ewing et al., 1998) was used to evaluate the quality of the electropherograms. Only sequences presenting Phred quality equal or higher than 20 were selected for generation of consensus sequences and downstream analyses (Ewing et al., 1998).

Next, the consensus sequences were subjected to BLASTn analysis to designate the molecular identity of the samples by comparing them with sequences previously deposited in the GenBank database (Benson et al., 2018). The sequences were aligned with other homologous sequences of the same gene using the Clustal W software via Bioedit v. 7.0.5.3 (Hall, 1999). The best nucleotide substitution model was determined by the software jModeltest v.2.1.10 (Darriba et al., 2012) through the PAUP* Version 4c software (Swofford, 2003). For Bayesian Inference, the software MrBayes 3.2.2 on XSEDE (Ronquist & Huelsenbeck, 2003) was used via the CIPRES (2025) portal.

### Genetic diversity analysis

The analysis of genetic diversity was performed using the sequences obtained from both the 16S rRNA and 23S rRNA genes. The calculation of nucleotide diversity (π), number of variable sites (VS), number of genotypes (g), genotype diversity (gd) and the average number of nucleotide differences (K) among the sequences obtained and closely related sequences previously deposited in GenBank (Benson et al., 2018) was done using the DnaSP v5 software (Librado & Rozas, 2009). A genotype network was constructed using the TCS v.1.21 (Clement et al., 2001), with figures generated using Population Analysis with Reticulate Trees software (PopART) (Leigh & Bryant, 2015). Additionally, a distance-based analysis was performed using SplitsTree V6.4.13 (University of Tubingen, Tubingen, Germany) and NeighborNet method (Huson & Bryant, 2006; Bryant & Huson, 2023) to investigate the genetic relationship among sequences in the present study and sequences previously deposited in GenBank and used in the genetic diversity analysis.

## Results

### PCR for the endogenous mammalian *gapdh* gene

Among 486 samples submitted to the mammalian endogenous *gapdh* gene, 94.44% (459/486) were positive: 80/80 from RO, 100/100 from GO, 147/155 from MG, and 132/151 from SP. The extracted DNA samples had a mean concentration and absorbance ratio (260/280 nm) of 30.3 ng/uL (ranging from 1.0 to 567.8 ng/μl, standard deviation [SD] ± 100.4) and 2.0 (ranging from 0.78 to 3.9, SD ± 0.42).

### Molecular detection of hemoplasmas using qPCR assays based on the 16S rRNA gene

In the qPCR assays, a positivity of 1.96% (9/459) was detected for hemoplasmas based on the 16S rRNA gene, previously positive in the endogenous *gapdh* gene: 1/80 from RO, 2/100 from GO, 5/147 from MG, and 1/132 from SP. The identification and origin of positive samples in the qPCR assays are shown in Table 2. The Efficiency, R^2^, Y-intercept and slope values ​​of the qPCR assays ranged from 97.9% to 103.8%, 0.983 to 0.997, 31.328 to 34.574, and -3.375 to -3.234, respectively, according to MIQE recommendations (Bustin et al., 2009). In the Melting temperature analysis, the positive samples corresponded to the Melting temperature (Tm) for ‘*Candidatus* Mycoplasma turicensis’ (Tm= 76 – 77.5 °C) (Willi et al., 2009) (Table 1).

**Table 2:** Positive samples according to the Melting temperature (Tm) obtained in the qPCR assay based on a fragment of the 16S rRNA gene with the respective quantification (number of DNA copies per microliter), and the Quantification Cycle (Cq) value.

**Sample identification (Origin)**	**Tm – Melting Temperature (°C) of the sample**	**Quantification (copies of a fragment of the 16S rRNA gene/μL)**	**Cq (Quantification Cycle)**
57 (São Paulo)	76.5	2.65 x 10^2^	11.49
51 (Minas Gerais)	77	2.75 x 10^1^	19.10
108 (Minas Gerais)	77.5	5.50 x 10^0^	21.51
116 (Minas Gerais)	77.5	6.35 x 102	17.86
22 (Minas Gerais)	77.5	*	*
36 (Minas Gerais)	76.5	*	*
39 (Goiás)	77.5	1.42 x 10^4^	20.1
104 (Goiás)	76.5	9.54 x 10^1^	27.75
I19 (Rondônia)	76.5	1.76 x 10^4^	21.1

* Non-quantifiable positive sample (differences between Cq values ​​greater than 0.5, even after triplicate repetition, probably due to the Monte Carlo effect - Bustin et al., 2009).

### Molecular characterization of hemoplasmas targeting the 16S rRNA and 23S rRNA genes

Of the nine samples submitted to molecular characterization, two (22.2% or 2/9) readable 16S rRNA gene sequences were obtained (both with 100% identity to ‘*Candidatus* Mycoplasma haemominutum’ [KR905457; EU839985.1]) from cats sampled in the states of Goiás and São Paulo, and three (33.3% or 3/9) sequences of the 23S rRNA gene were obtained in the states of Minas Gerais (99.85% identity to ‘*Candidatus* Mycoplasma haemominutum’ [HE613254.1]), São Paulo (99.85% of identity to ‘*Candidatus* Mycoplasma haemominutum’ [HE613254.1]) and Goiás (99.21% identity to ‘*Candidatus* Mycoplasma haemominutum’ [HE613254.1]). All sequences have been deposited in GenBank under accession numbers OR381484, PP809395, OR738640, PP691102 and PP669273.

### Phylogenetic analyzes based on the 16S rRNA and 23S rRNA genes

**Figure 2:**
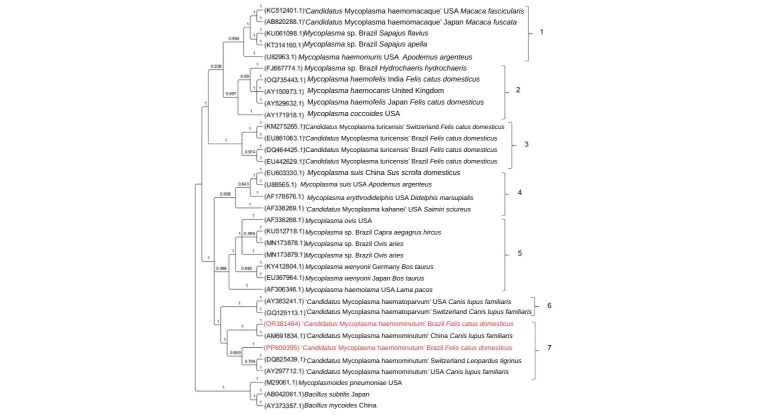
Phylogenetic tree based on an alignment of 989 bp length of *Mycoplasma* spp. 16S rRNA sequences, created using the Bayesian method, and GTR+I+G as the evolutionary model. The sequences obtained in this study are highlighted in red. *Mycoplasmoides pneumoniae*, *Bacillus subtilis* and *Bacillus mycoides* were used as an outgroup.

The Bayesian inference (GTR+I+G evolutionary model) of 35 homologous sequences of the 16S rRNA gene (989 bp alignment) showed the formation of two large distinct clades, which are subdivided into seven smaller clades (Figure 2). The eight subclades were numbered from one to seven in the phylogenetic tree represented in Figure 2. In the seventh clade, five sequences from ‘*Candidatus* Mycoplasma haemominutum’ were grouped, together with the sequences obtained in the present study obtained from two domestic cats in Brazil (PP809395 from São Paulo; OR381484 from Goiás). Among the ‘*Candidatus* Mycoplasma haemominutum’ sequences allocated in subclade 7, one sequence was detected in a dog (*Canis lupus familiaris*) from China (AM691834) and another in a dog from the United States (AY297712), as well as a sequence detected in a margay cat (*Leopardus tigrinus*) from Switzerland (DQ825439). Two sequences of ‘*Candidatus* Mycoplasma haematoparvum’ obtained in dogs (*Canis lupus familiaris*) were also grouped, one from the United States (AY383241) and one from Switzerland (GQ129113) in the subclade 6. The *Mycoplasma haemofelis* and ‘*Candidatus* Mycoplasma turicensis’ sequences were positioned in subclades 2 and 3, respectively, remaining distant from the sequences obtained in the present study.

**Figure 3:**
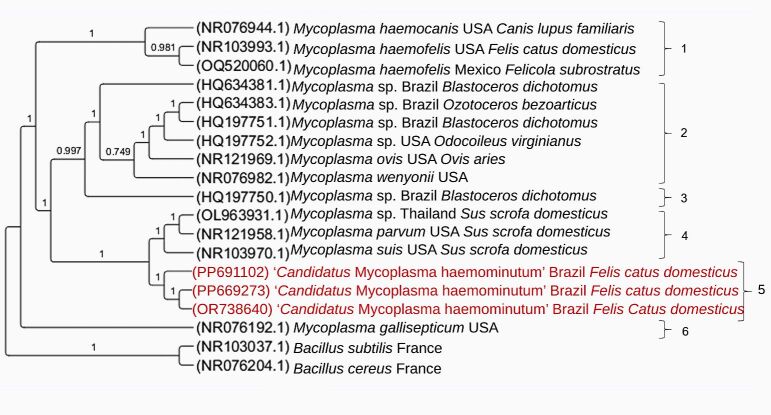
Phylogenetic tree based on an alignment of 759 bp length of *Mycoplasma* spp. 23S rRNA sequences, created using the Bayesian method and GTR+G as the evolutionary model. The sequences obtained in this study are highlighted in red. *Bacillus cereus* and *Bacillus subtilis* were used as an outgroup.

The Bayesian inference (GTR+I+G evolutionary model) of 21 homologous sequences of the 23S rRNA gene (759 bp alignment) showed the formation of two large distinct clades, which were subdivided into six smaller clades (Figure 3). The six subclades were numbered from one to six in the phylogenetic tree represented in Figure 3. In the subclade 5, the three sequences obtained in the present study (PP669273 from Minas Gerais, PP691102 from São Paulo, and OR738640 from Goiás) of ‘*Candidatus* Mycoplasma haemominutum’ from naturally infected domestic cats in Brazil were positioned together in a sister subclade to sequences of hemoplasma species detected in pigs (OL963931 [*Mycoplasma* sp], NR121958 [*Mycoplasma parvum*], and NR103970 [*Mycoplasma suis*]) from Thailand and the United States of America).

### Genetic diversity analysis

**Figure 4:**
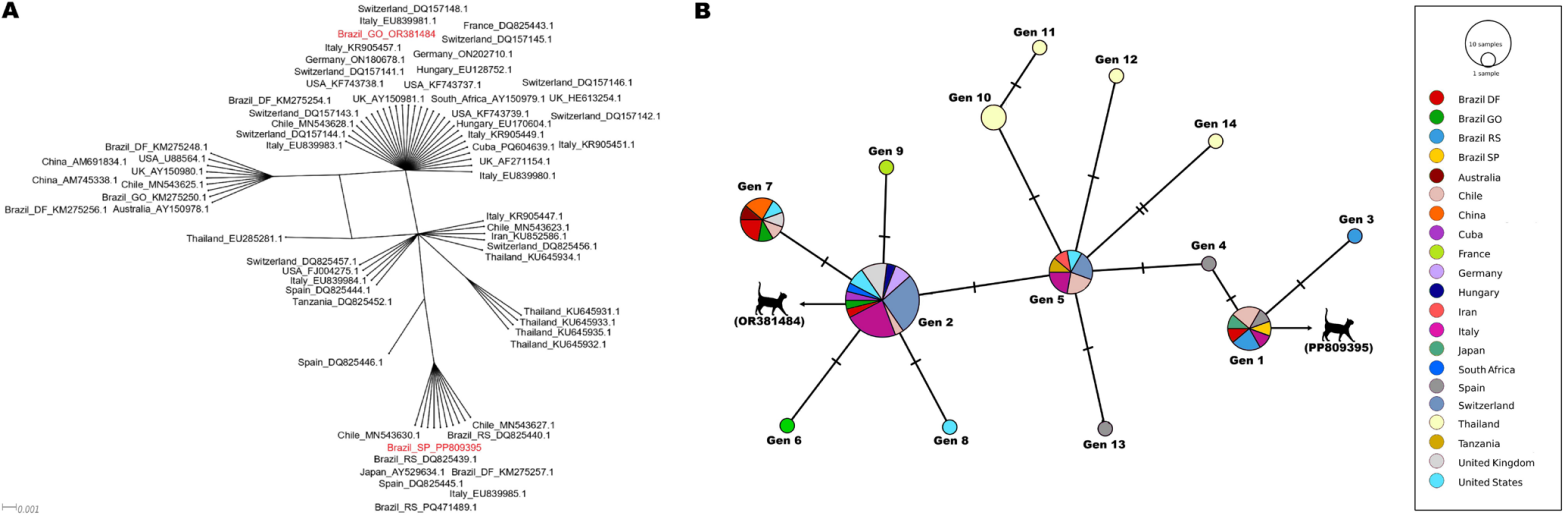
(A) ‘*Candidatus* Mycoplasma haemominutum’ split-network distance analysis of the 16S rRNA sequences. The analysis was performed applying the NeighborNet method based on a 469 bp alignment. The sequences obtained in the present study are highlighted in red; (B) Genotype network obtained from 16S rRNA sequences (469 bp) of *'Candidatus* Mycoplasma haemominutum' detected in domestic cats, wild felids and a dog worldwide. The size of the circles varies according to the number of sequences comprising each genotype. Each color represents the location where each sequence was sampled. The black vertical lines represent mutational events between each genotype. The 16S rRNA genotypes obtained in the present study are represented by an illustration of a cat (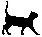), followed by the accession number on GenBank.

**Figure 5:**
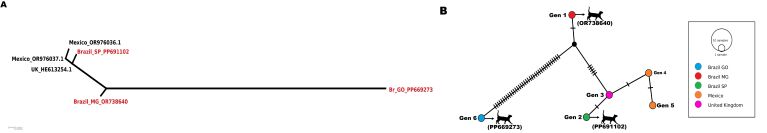
(A) ‘*Candidatus* Mycoplasma haemominutum’ split-network distance analysis of the 23S rRNA sequences. The analysis was performed applying the NeighborNet method based on a 671 bp alignment. The sequences obtained in the present study are highlighted in red; (B) Genotype network obtained from 23S rRNA sequences (671 bp) of *'Candidatus* Mycoplasma haemominutum' detected in domestic cats and humans worldwide. The size of the circles varies according to the number of sequences from each genotype. Each color represents the location where each sequence was sampled. The black vertical lines represent mutational events between each genotype. The 23S rRNA genotypes obtained in the present study are represented by an illustration of a cat (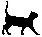), followed by the accession number on GenBank.

The SplitsTree distance-based analyses were constructed based on the same alignments used in the genetic diversity analyses for the 16S rRNA (469 bp) and 23S rRNA (671 bp) genes, containing the sequences detected in the present study and closely related ‘*Candidatus* Mycoplasma haemominutum’ sequences from GenBank (Figures 4A and 5A).

16S rRNA sequences detected in domestic cats from the states of GO and SP represented two different genotypes (Figure 4): while the sequence OR381484 (GO) corresponded to genotype #1, the sequence PP809395 (SP) corresponded to genotype #2. The number of sequences analyzed, variable sites number, G+C content, genotypes number, genotype diversity, standard deviation, nucleotide diversity (per site) and nucleotide differences number are shown in Table 3.

**Table 3:** Results of genotypic diversity analysis.

**Gene**	**bp**	**N**	**VS**	**GC%**		**g**	**gd (mean ± SD)**	**π (mean ± SD)**	**K**
16S rRNA	470	65	14	43.1	16		0.790±0.038	0.00363± 0.00035	1.675
23S rRNA	667	6	372	39.3	6		0.00926±0.096	0.0118872±0.10903	131.33333

N= Number of sequences analyzed; VS= number of variable sites; GC%= G+C content; g= number of genotypes; gd= genotype diversity; SD= standard deviation; π= nucleotide diversity (per site); K= number of nucleotide differences.

23S rRNA sequences detected in domestic cats from the states of GO, SP, and MG represented three different genotypes (Figure 5): while the sequence PP669273 (GO) corresponded to genotype #6, the sequence PP691102 (SP) corresponded to genotype #2, and OR738640 (MG) corresponded to genotype #1. The number of sequences analyzed, variable sites number, G+C content, genotypes number, genotype diversity, standard deviation, nucleotide diversity (per site) and nucleotide differences number are shown in Table 3.

## Discussion

In the present study, molecular techniques were used to detect the occurrence of hemoplasmas in domestic cats from different veterinary clinics, autonomous animal protection shelters and castration campaigns in the states of Goiás, São Paulo, Minas Gerais and Rondônia. Among the 459 samples submitted to qPCR assays based on the 16S rRNA gene for detection of hemotropic mycoplasmas, 1.96% (9/459) were positive, making this the first study to describe the occurrence of such agents in the states of Rondônia and Minas Gerais, to the best of our knowledge.

The molecular occurrence of hemoplasmas varies between regions of Brazil. In the South, detection varies between 6.6 and 21.4% (Pedrassani et al., 2019; Santis et al., 2014), 6.5 to 32% in the Southeast (André et al., 2014; Bortoli et al., 2012; Raimundo et al., 2016), 8.4 to 36.4% in the Central-West (Miceli et al., 2013; Santis et al., 2014, Carvalho et al., 2024), 12 to 35.5% in the Northeast (Braga et al., 2012; Munhoz et al., 2018), and 44% in the North (Santos et al., 2021). Although the present study used qPCR to detect hemoplasmas, which is a more sensitive technique compared to PCR, the frequency of hemoplasma DNA detected was considered low, but similar to other studies carried out in Brazil. Miceli et al. (2013) detected 8.4% positivity for hemoplasmas in blood samples from naturally infected cats in the city of Cuiabá (MT), using conventional PCR assays. Likewise, Bortoli et al. (2012) and Pedrassani et al. (2019) reported frequencies corresponding to 6.5% and 6.6% in the states of São Paulo and Santa Catarina, respectively. The variation in the molecular occurrence of hemoplasmas in domestic cats between different regions of the country can be influenced by environmental and climatic conditions, veterinary care, characteristics of the sampled feline population (housed versus non-housed cats), immune status of the sampled animal, sample size, and sensitivity of the molecular assay used in the diagnosis of infection by such agents (Imre et al., 2020; Mesquita et al., 2021; Hoseinpoor et al., 2024).

Although there are reports of co-infections between two or three hemoplasma species, the present work detected only CMhm in nine (9/459) naturally infected cats (Tasker, 2022; Hoseinpoor et al., 2024). Furthermore, CMhm is the species most frequently detected in domestic cats in Brazil and other countries, corroborating the findings of this study (Santos et al., 2021; Munhoz et al., 2018; Santis et al., 2014; Imre et al., 2020; Mesquita et al., 2021; Hoseinpoor et al., 2024). Such observations are associated with CMhm’s high efficiency in infecting and multiplying in cats compared with Mhf and CMt (Tanahara et al., 2010; Hoseinpoor et al., 2024).

Although the 16S rRNA gene is highly conserved, bacterial species present considerable variations in this molecular marker, allowing differentiation between them. Based on this, the 16S rRNA gene has been extensively used in PCR assays to detect and differentiate hemoplasma species through different primer pairs, including those used in the present study (Martinez-Porchas et al., 2017; Citti et al., 2020). Although the Melting curve analysis of the positive samples in this study corresponded molecularly to CMt (Tm= 76.5 °C and 77.5 °C), the semi-nested PCR characterization assays based on the 16S rRNA gene and conventional PCR based on the 23S rRNA gene, followed by sequencing and BLASTn analysis, confirmed the molecular identity of CMhm, indicating a possible mistake in the Melting temperatures established in the protocol by Willi et al. (2009). These findings were corroborated with phylogenetic analyses based on both 16S rRNA and 23S rRNA, which positioned the sequences detected in this study with others from CMhm and separately from CMt sequences.

In the phylogenetic analysis, the 16S rRNA sequences obtained in this study (OR381484 and PP809395) were positioned within the clade of ‘*Candidatus* Mycoplasma haemominutum*’* (CMhm), clustering with sequences from different geographic regions, such as Brazil, Switzerland, China, and the United States, supported by high statistical values. This result reinforces the low genetic diversity commonly reported for CMhm worldwide when using this molecular marker and suggests the existence of genotypes with a wide geographic distribution (Imre et al., 2020). In addition, the genotype network analysis demonstrated that PP809395 and OR381484 were represented by two distinct genotypes (Genotypes #1 and #2, respectively), distant from each other by a few mutational events and two genotypes (#4 and #5). The grouping of these genotypes with others from different countries may evidence limited global 16S rRNA gene flow of CMhm. Nonetheless, the presence of exclusive genotypes in Brazilian samples (genotypes #1 and #2) suggests local variations and possible regional adaptations, despite the overall low diversity observed. These findings might suggest recent population expansion or transmission of CMhm among domestic cats, characterized by low selective pressure and limited accumulated genetic diversity. Although CMhm is classically associated with subclinical infections in immunocompetent cats, the results of the present study are epidemiologically and clinically relevant, as this agent can cause hemolytic anemia, particularly in immunosuppressed animals or those co-infected with other pathogens, such as feline immunodeficiency virus (FIV), feline leukemia virus (FeLV) or other hemoplasma and hemoparasite species (Rosa Maciel et al., 2023).

A robust genetic diversity analysis of CMhm 23S rRNA was not feasible due to the limited number of sequences available in public databases. Another limitation of this study was the lack of information related to the clinical history of the sampled animals, especially if they had been recently treated with antibiotics capable of controlling hemoplasma infection, such as doxycycline and enrofloxacin. The inclusion of hematological and biochemical profiles, as well as the investigation of the association between hemoplasma infection and feline immunodeficiency virus (FIV) and feline leukemia virus (FeLV) infections should be included in future studies. Additionally, considering that sampling was performed by convenience, the real prevalence of these hemotropic agents should be cautiously interpreted.

## Conclusion

This is the first molecular detection of hemoplasmas in cat blood samples in the states of Minas Gerais and Rondônia. Furthermore, the identification of two genotypes with wide global distribution circulating in Brazil contributes to a better understanding of the molecular epidemiology of feline hemoplasmosis agents in Brazil. The presence of exclusive genotypes in Brazilian samples suggests local variations and possible regional adaptations, despite the overall low diversity observed.

## Data Availability

Data will be made available on request.
